# Supplementary material for: Analysis of the Genetic Diversity of Two Rhopalosiphum Species from China and Europe Based on Nuclear and Mitochondrial Genes
Source: Insects. 2023 Jan 6;14(1):57. doi: 10.3390/insects14010057 (PMC9866154; doi:10.3390/insects14010057)
Supplement: Supplementary file 1 [file insects-14-00057-s001.zip › insects-2062384-Supplementary.pdf]

Analysis of the genetic diversity of two *Rhopalosiphum* aphids from China and European countries based on one nuclear and two mitochondrial genes

Table S1. Sampling information of *R. padi* individuals investigated

Corn Region	Province	Index	Population	Locality	Geo-coordinates	Date	Number
**North spring maize region (NS)**	Heilongjiang	1	HEB	Haerbin	45°49′ N, 126°48′ E	14 Aug. 2014	14
		2	HBP	Haerbin	45°38′ N, 126°38′ E	24 Jul. 2016	22
		3	HBD	Haerbin	45°50′ N, 126°50′ E	24 Jul. 2016	16
		4	HG	Hegang	47°8′ N, 130°17′ E	5 Aug. 2014	17
		5	SYS	Shuangyashan	46°46′ N, 131°06′ E	7 Aug. 2014	2
	Jilin	6	TH	Tonghua	42°22′ N, 125°25′ E	1 Aug. 2014	19
	Liaoning	7	SY	Shenyang	41°49′ N, 123°33′ E	28 Jul. 2014	18
	Inner Mongolia	8	TL	Tongliao	43°40′ N, 122°21′ E	11 Aug. 2014	23
		9	TMT	Tumd Right Banner	40°36′ N, 110°34′ E	4 Sep. 2016	24
	Ningxia	10	NX	Qingtongxia	38°1′ N, 106°4′ E	4 Aug. 2016	18
	Gansu	11	ZY	Zhangye	38°56′ N, 100°27′ E	30 Aug. 2016	18
	Hebei	12	ZJK	Zhangjiakou	40°44′ N, 114°52′ E	21 Aug. 2014	23
	Shanxi	13	XZ	Xinzhou	38°25′ N, 112°43′ E	26 Aug. 2014	14
	Shaanxi	14	YuL	Yulin	38°20′ N, 109°46′ E	28 Aug. 2014	24
**Huanghuaihai summer maize region (HS)**	Hebei	15	HD	Handan	36°56′ N, 114°52′ E	27 Aug. 2014	14
		16	LF	Langfang	39°28′ N, 116°38′ E	31 Aug. 2014	20
	Shandong	17	JNi	Jining	35°5′ N, 116°34′ E	4 Sep. 2014	12
		18	WF	Weifang	36°54′ N, 119°10′ E	3 Sep. 2014	11
	Henan	19	XX	Xinxiang	35°18′ N, 113°53′ E	15 Sep. 2014	8
	Beijing	20	BJ	Beijing	40°1′ N, 116°16′ E	19 Aug. 2014	22
	Anhui	21	SZ	Suzhou	33°38′ N, 117°4′ E	19 Sep. 2014	23
	Shaanxi	22	YaL	Yangling	34°16′ N, 108°3′ E	23 Sep. 2014	11
**Southwest hilly maize region (SWH)**	Yunnan	23	MS	Mangshi	24°26′ N, 98°35′ E	24 Aug. 2014	20
	Sichuan	24	MZ	Mianzhu	31°24′ N, 104°18′ E	5 Jul. 2016	14
	Guizhou	25	GY	Guiyang	26°30′ N, 106°39′ E	8 Aug. 2016	19
**Northwest inland maize region (NWI)**	Xinjiang	26	YN	Yining	43°59′ N, 81°32′ E	14 Aug. 2014	18
		27	KS	Kashi	39°28′ N, 75°59′ E	14 Aug. 2014	6
		28	QT	Qitai	44°4′ N, 89°44′ E	26 Aug. 2016	17
**European countries (EUR)**	Belgium	29	DN	Dinant	50°34′ N, 4°41′ E	26 Sep. 2015	14
	Luxembourg	30	LSB	Alzingen	49°34′ N, 6°9′ E	28 Sep. 2015	4
	France	31	FR	Strasbourg	48°38′ N, 7°37′ E	2 Oct. 2015	27
	Germany	32	GM	Ingolstadt	48°44′ N, 11°25′ E	4 Oct. 2015	23

Table S2. Sampling information of *R. maidis* individuals investigated

Corn Region	Province	Index	Population	Locality	Geo-coordinates	Date	Number
**North spring maize region (NS)**	Heilongjiang	1	HEB	Haerbin	45°49′ N, 126°48′ E	16 Aug. 2014	23
	Jilin	2	GZL	Gongzhuling	43°31′ N, 124°48′E	4 Sep. 2014	24
	Liaoning	3	SY	Shenyang	41°49′ N, 123°33′ E	28 Aug. 2014	23
	Inner	4	TL	Tongliao	43°40′ N, 122°21′ E	11 Aug. 2014	18
	Mongolia	5	TMT	Tumd Right Banner	40°36′ N, 110°34′ E	4 Sep. 2016	9
	Hebei	6	ZJK	Zhangjiakou	40°45′ N, 114°53′ E	21 Aug. 2014	22
		7	LP	Luanping	40°56′ N, 117°19′ E	4 Sep. 2015	22
	Shanxi	8	XZ	Xinzhou	38°25′ N, 112°44′ E	26 Aug. 2014	21
	Shaanxi	9	YuL	Yulin	38°20′ N, 109°46′ E	28 Aug. 2014	31
	Gansu	10	ZY	Zhangye	38°51′ N, 100°34′ E	24 Aug. 2014	18
		11	TS	Tianshui	34°44′ N, 105°20′ E	2 Sep. 2016	21
		12	PL	Pingliang	35°20′ N, 107°22′ E	24 Aug. 2016	10
**Huanghuaihai summer maize region (HS)**	Hebei	13	HS	Hengshui	37°43′ N, 115°44′ E	20 Aug. 2014	23
		14	HD	Handan	36°56′ N, 114°52′ E	27 Aug. 2014	18
		15	SJZ	Shijianzhuang	37°54′ N, 114°37′ E	28 Aug. 2014	24
		16	LF	Langfang	39°29′ N, 116°38′ E	31 Aug. 2014	23
	Shandong	17	JNi	Jining	35°5′ N, 116°34′ E	4 Sep. 2014	23
		18	DZ	Dezhou	37°28′ N, 116°19′ E	25 Aug. 2014	12
		19	WF	Weifang	36°54′ N, 119°10′ E	3 Sep. 2014	23
		20	JNa	Jinan	36°46′ N, 117°31′ E	5 Sep. 2014	11
	Henan	21	LY	Luoyang	34°38′ N, 112°29′ E	22 Sep. 2014	21
		22	XX	Xinxiang	35°18′ N, 113°53′ E	15 Sep. 2014	23
		23	LH	Luohe	33°35′ N, 114°1′ E	17 Sep. 2014	23
	Beijing	24	BJ	Beijing	40°2′ N, 116°16′ E	6 Aug. 2014	20
	Anhui	25	SZ	Suzhou	33°38′ N, 117°4′ E	19 Sep. 2014	22
		26	HF	Hefei	30°24′ N, 116°59′ E	17 Oct. 2014	24
	Shaanxi	27	YaL	Yangling	34°17′ N, 108°3′ E	23 Sep. 2014	21
**Southwest hilly maize region (SWH)**	Sichuan	28	XD	Xindu	30°47′ N, 104°13′ E	12 Aug. 2014	21
		29	MZ	Mianzhu	31°24′ N, 104°18′ E	28 Aug. 2014	24
		30	NC	Nanchong	30°53′ N, 106°3′ E	3 Aug. 2016	22
	Chongqing	31	CQ	Chongqing	29°29′ N, 106°22′ E	6 Aug. 2016	34
	Guizhou	32	GY	Guiyang	26°30′ N, 106°39′ E	8 Aug. 2016	29
	Yunnan	33	MS	Mangshi	24°26′ N, 98°35′ E	20 Sep. 2014	26
**Southeast hilly maize region (SEH)**	Zhejiang	34	DY	Dongyang	29°27′ N, 120°32′ E	20 Sep. 2014	22
	Hunan	35	CS	Changsha	28°12′ N, 113°05′ E	14 Sep. 2014	9
	Guangdong	36	GZ	Guangzhou	23°09′ N, 113°21′ E	8 Nov. 2014	23
	Hainan	37	YC	Yacheng	18°24′ N, 109°12′ E	17 Jan. 2016	29
**France (FRA)**	Hérault	38	FR	Montpellier	43°37′ N, 3°53′ E	20 Sep. 2015	16

Table S3. PCR primers used in this study

**Aphid species**	**Gene name**	**Primer sequence (5'-3')**	**Annealing temperature**	**Product size (bp)**	**Accession numbers in Genbank**
Rhopalosiphum padi	COI	F: GATCAGGTATAATTGGTTCATC	54℃	597	DQ499056
		R: CCTCAGGGTCAAAGAATGATG			
	COII	F: CTTGAATAAAACTAAGATTTCAAAAT	52℃	615	U36749
		R: GAATAGGTATAAATCTGTGATTAAT			
	EF-1α	F: TCACCATCATTGACGCACC	52℃	765	AY219719
		R: GTGCATCTCTACGGACTTAACTTC			
Rhopalosiphum maidis	COI	F: CTTACTGATCGAAATTTAAATACT	50℃	681	JQ860264
		R: CATACCATTTAAACCTAAAAAATGT			
	COII	F: CTTGATTAAAACTAAGATTTCAAAAT	50℃	615	AY219738
		R: GAATAGGTATAAATCTATGATTAAT			
	EF-1α	F: ATGGACAAACCCGTGAA	50℃	784	JQ860288
		R: TTACCTGGGCTGTGAAAT			

Table S4. Variable positions of 29 haplotypes of mtDNA gene sequences for *R. padi*

	Nucleotide positions of the combined COI and COII sequences (bp)																															
	7	8	1	1	1	1	1	2	2	2	2	3	3	3	4	4	4	4	5	5	5	6	6	6	7	7	7	8	8	8	9	9
H	8	5	0	2	5	5	5	0	3	8	9	0	2	7	1	2	3	5	5	6	9	3	3	9	0	2	5	5	5	5	4	7
			2	3	1	6	9	1	1	8	8	3	1	8	5	0	5	5	0	5	9	4	8	4	6	5	5	0	4	6	0	3
H1	A	T	A	C	T	A	T	C	C	T	G	A	A	T	G	A	C	C	T	T	C	T	C	T	C	G	A	G	G	C	T	C
H2														C																		
H3														C			T															
H4		C		T	C		C		T				G	C			T					A	T					A		T		
H5	G	C		T	C		C	T	T				G	C			T					A	T					A		T		
H6	G	C		T	C		C		T				G	C			T					A	T					A		T		
H7		C		T	C		C		T				G	C			T					A	T			A		A		T		
H8	G	C		T	C		C						G	C			T					A	T					A		T		

H9	G	C		T	C		C		T				G	C			T		C			A	T					A		T		
H10		C		T	C		C		T				G	C			T					A	T	C				A		T		
H11	G	C	G	T	C		C		T				G	C			T					A	T					A		T		
H12		C		T	C		C		T				G	C			T	T				A	T					A		T		
H13														C			T				T											
H14														C															A			
H15														C			T											A				
H16												G		C			T															
H17														C			T										G					
H18																				C												
H19																												A				
H20														C														A				
H21																																T
H22														C			T					C										
H23						G																										
H24														C	A		T															
H25										C				C			T															
H26																									T							
H27																															C	
H28											A			C			T															
H29																T																

H, haplotypes; blank means nucleotide is the same to H1.

Table S5. Variable positions of 32 haplotypes of EF-1α gene sequences for *R. padi*

	Nucleotide positions of EF-1α sequence (bp)																				
H	1	1	3	5	6	9	1	2	2	2	2	3	4	5	5	5	5	6	6	6	6	6
	2	5	6	1	9	0	5	0	0	1	2	0	3	1	1	2	9	1	2	3	3	4
							6	4	6	1	5	6	3	0	4	3	9	7	8	1	8	7
H1	C	C	C	T	C	C	C	C	A	G	G	C	G	G	T	C	G	C	C	T	G	G
H2				C																		
H3			T																		A	
H4											A											
H5														A								
H6																						T
H7			T								A											
H8			T	C																		
H9			T																			
H10								T														
H11				C				T														
H12				C							A											
H13																T						
H14				C														T				
H15													T									
H16																				A		
H17											A			A								
H18				C	T																	
H19			T	C														T				
H20				C					T													
H21											A								T			
H22																	A					
H23																		T				
H24																			T			

H25		T		C															
H26	T		T									T							
H27				C															T
H28							T												
H29										T					A				
H30												T							
H31						T													
H32			T					T											

H, haplotypes; blank means nucleotide is the same to H1.

Table S6. Variable positions of 7 haplotypes of EF-1α gene sequences for *R. maidis*

	Nucleotide positions of EF-1α sequence (bp)			
H	22	222	550	610
H1	T	C	T	C
H2		T		
H3	A			
H4			C	
H5	A		C	
H6			C	T
H7				T

H, haplotypes; blank means nucleotide is the same to H1.

Table S7. Variable positions of 12 haplotypes of mtDNA gene sequences for *R. maidis*

	Nucleotide positions of the combined COI and COII sequences (bp)										
H	282	474	501	549	555	743	913	925	928	982	1018
H1	T	T	T	T	T	C	A	T	T	A	A
H2											G
H3				C							
H4							G				

H5						T				
H6		C								
H7										G
H8			C							
H9								G		
H10									C	
H11					C					
H12	A									

H, haplotypes; blank means nucleotide is the same to H1.

Table S8. AMOVA analysis among 32 *R. padi* and 38 *R. maidis* populations according to their geographic locations

Aphid species	Genes	Source of variation	d.f.	Sum of squares	Variance components	Percentage of variation	F_{ST}
R. padi	COI	Among	31	76.292	0.13647 Va	41.93	0.419 ***
		Within	503	95.059	0.18898 Vb	58.07	
	COII	Among	31	55.422	0.10476 Va	70.55	0.706 ***
		Within	503	21.995	0.04373 Vb	29.45	
	COI- COII	Among	31	72.426	0.12785 Va	38.09	0.381 ***
		Within	503	104.535	0.20782 Vb	61.91	
	EF-1α	Among	31	28.534	0.04252 Va	16.67	0.167 ***
		Within	503	106.935	0.21259 Vb	83.33	
R. maidis	COI	Among	37	0.914	-0.00004 Va	-0.16	-0.002
		Within	770	19.695	0.02558 Vb	100.16	
	COII	Among	37	9.157	0.00775 Va	8.53	0.085 ***
		Within	770	63.979	0.08309 Vb	91.47	
	COI- COII	Among	37	9.764	0.00742 Va	6.52	0.065 ***
		Within	770	81.902	0.10637 Vb	93.48	
	EF-1α	Among	37	19.517	0.01251 Va	4.56	0.046 ***

Within	770	201.668	0.26191 Vb	95.44

Among, among populations; Within, within populations; d.f., degrees of freedom; *** P < 0.001.

Table S9. AMOVA analysis among 5 groups of *R. padi* and *R. maidis* according to their geographic locations based on the combined mtDNA and nuclear EF-1α

Aphid species	Genes	Source of variation	d.f.	Sum of squares	Variance components	Percentage of variation	Fixation indices
R. padi	COI- COII	Among groups	4	46.562	0.11394 Va	31.00	F_{CT} = 0.310 ***
		Within groups	27	25.864	0.04574 Vb	12.45	F_{SC} = 0.180 ***
		Within populations	503	104.535	0.20782 Vc	56.56	F_{ST} = 0.435 ***
		Total	534	176.961	0.36751		
	EF-1α	Among groups	4	10.252	0.01969 Va	7.56	F_{CT} = 0.076 **
		Within groups	27	18.282	0.02833 Vb	10.87	F_{SC} = 0.118 ***
		Within populations	503	106.935	0.21259 Vc	81.57	F_{ST} = 0.184 ***
		Total	534	135.469	0.26061		
R. maidis	COI- COII	Among groups	4	1,826	0.00148 Va	1.29	F_{CT} = 0.013
		Within groups	33	7.938	0.00634 Vb	5.55	F_{SC} = 0.056 ***
		Within populations	770	81.902	0.10637 Vc	93.16	F_{ST} = 0.068 ***
		Total	807	91.666	0.11418		
	EF-1α	Among groups	4	4.531	0.00467 Va	1.70	F_{CT} = 0.017
		Within groups	33	14.986	0.00908 Vb	3.29	F_{SC} = 0.034 **
		Within populations	770	201.668	0.26191 Vc	95.01	F_{ST} = 0.050 ***
		Total	807	221.186	0.27566		

d.f., degrees of freedom; * P < 0.05, ** P < 0.01, *** P < 0.001.
